# Datawiz-IN: fostering representative innovation in health data science—outcomes from a summer research experience

**DOI:** 10.1186/s12909-025-07298-1

**Published:** 2025-05-28

**Authors:** Sadia Afreen, Alexander Krohannon, Saptarshi Purkayastha, Sarath Chandra Janga

**Affiliations:** Department of Biomedical Engineering and Informatics, Indiana University Indianapolis, Indianapolis, IN 46202 USA

**Keywords:** Biomedical AI; Data Science Training, Representative Innovation, Biomedical Education, Summer Research Experience

## Abstract

The growing adoption of Artificial Intelligence (AI) across sectors highlights the importance of diverse perspectives in guiding its development and implementation. This study examines”Datawiz-IN” an educational initiative that provides data science and machine learning research experience to students from various backgrounds in biomedicine. Supported by a National Institutes of Health R25 grant from the National Library of Medicine, the program engaged cohorts of 14 students in Summer 2023 and 13 students in Summer 2024. Initial data suggest modest increases in representation, with higher participation rates of women and less prevalant students compared to typical AI research programs. Student projects addressed various aspects of biomedical data science, including disease mechanism analysis, clinical decision support systems, and health disparity investigations. While the program’s limited scale and short duration constrain broad generalizations, preliminary results indicate the potential benefits of structured inclusion efforts in expanding participation in AI research and development. This case study contributes to ongoing discussions about approaches for developing more representative AI systems and research communities, though longer-term studies will be needed to assess sustained impact. The findings suggest that targeted educational initiatives may play a role in broadening participation in AI development, while acknowledging that meaningful change requires sustained, systemic efforts across multiple institutions and career stages.

**Supplementary Information:**

The online version contains supplementary material available at 10.1186/s12909-025-07298-1.

## Introduction

Artificial intelligence (AI) adoption is rapidly expanding across sectors, yet balanced access remain elusive [1]. Further, AI systems may unintentionally perpetuate variability, yielding marginalized outcomes, particularly in healthcare applications [2]. Varied perspectives must inform AI ethics and governance to mitigate such risks, especially as these systems increasingly influence critical healthcare decisions. Currently, AI guidelines and regulations disproportionately reflect the viewpoints of industrialized nations, failing to account for the distinct values of less developed regions [3, 4]. These homogeneous perspectives risk perpetuating variability and limiting societal benefits [1, 5].

The underrepresentation of underrepresented voices in AI development is particularly concerning, with women and racial minorities comprising only 10–15% of the AI field [6]. This disparity reflects longstanding representation gaps in technology-related disciplines [7] and has direct implications for healthcare AI development and deployment.

**Fig. 1:**
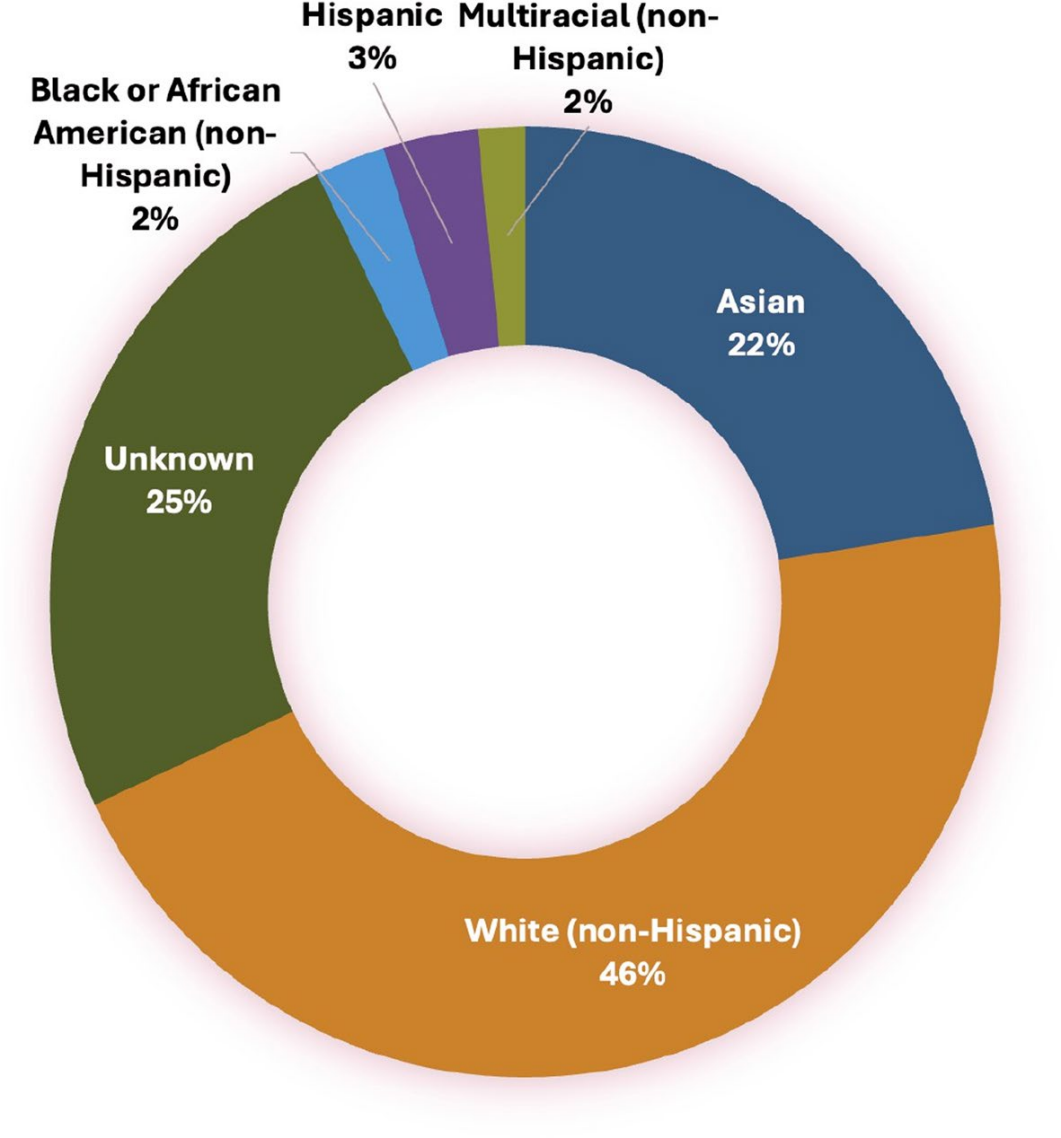
Ethnic representation across AI [8]

**Fig. 2:**
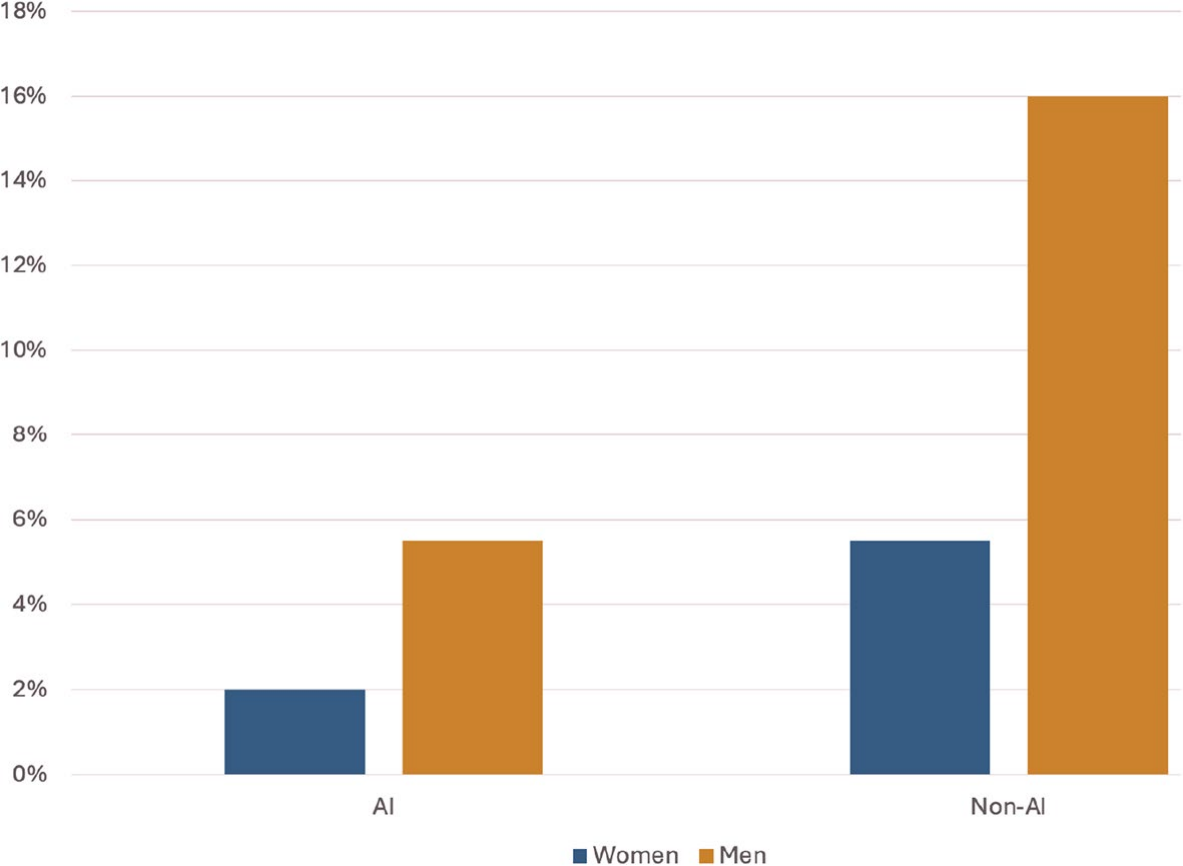
Gender representation across AI

Research demonstrates that representation spurs innovation, corrects variability, and promotes user-centric design [9], making open participation crucial for advancing access-driven AI solutions [10] (Figs 1 and 2).

**Fig. 3:**
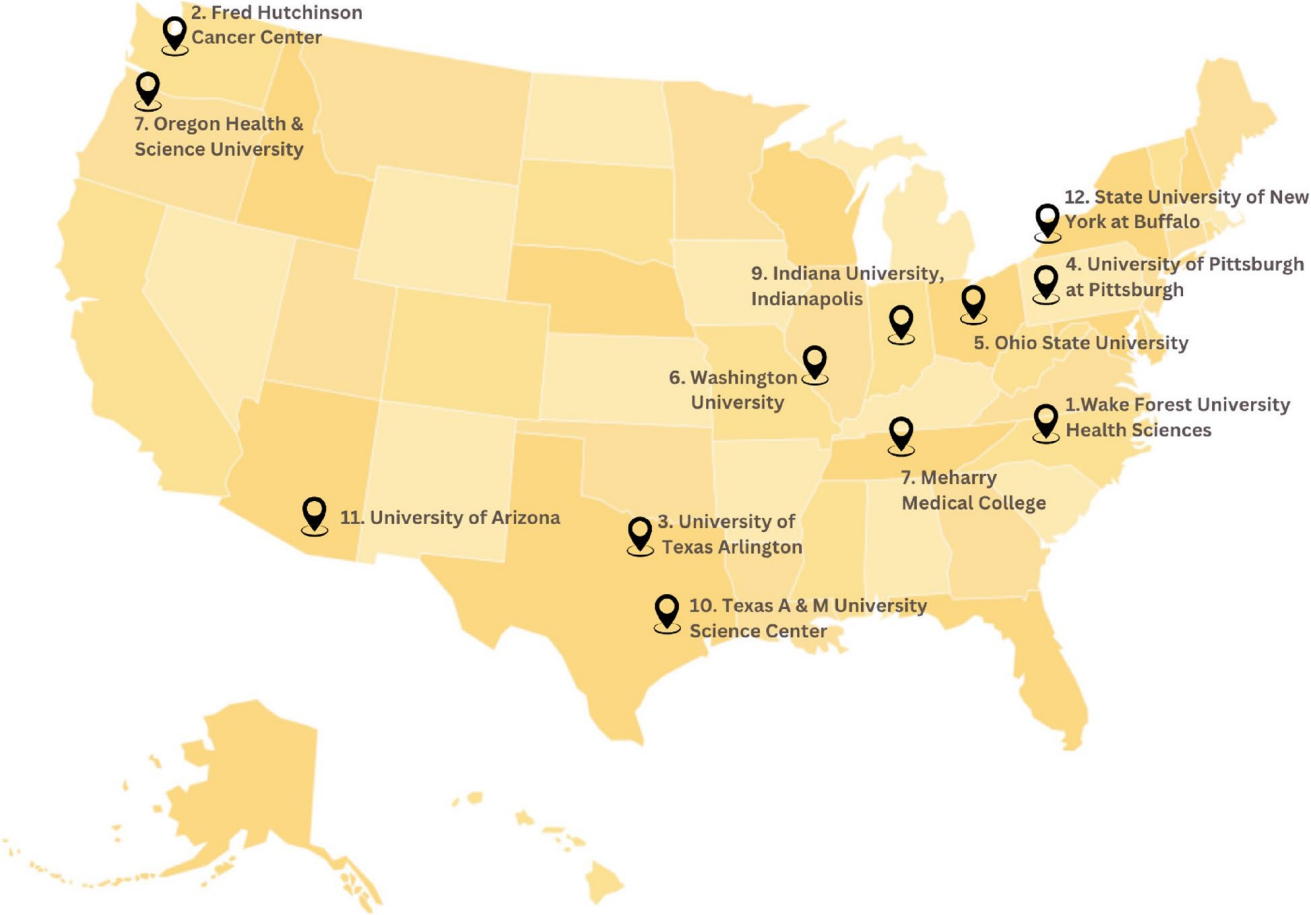
Spread of NLM R25 initiative across various institutions demonstrates the nationwide commitment to diversifying biomedical informatics training

Several initiatives have emerged to address this gap in AI diversity. While programs like AI4 ALL have achieved 40% women participation [11] and Carnegie Mellon University’s Data Science for All program [12] provides data literacy training, few focus specifically on healthcare AI applications. Recognizing this need, the National Institutes of Health (NIH) established the R25 initiative to support biomedical informatics training for historically excluded groups. This initiative provides targeted grants for specialized training programs developing hands-on expertise, rather than broad frameworks, to confront marginalization in biomedicine. As summarized by the NLM (2019), these programs actively strive to rectify representation gaps and power impactful research by students from underrepresented backgrounds. Through research training collaborations across over 30 higher education institutions (Fig. 3), the R25 initiative has created a nationwide network supporting diversity in biomedical informatics [13].

To address this critical need, we developed Datawiz-IN in 2023, building upon.

Indiana University’s long-standing commitment to diversity in STEM education. The program leverages the established Indiana University-Minority Serving Institutions (IU-MSI) STEM Initiative, which since 2006 has fostered partnerships with Historically Black Colleges and Universities (HBCUs), Hispanic-serving institutions, and Tribal Colleges [14]. Through these strategic collaborations, Datawiz-IN specifically aims to increase the participation of students from marginalized backgrounds in healthcare AI development.

The program represents a targeted response to the “leaky academic pipeline” [15] that has historically limited diversity in advanced scientific fields. Through NIH R25 funding, Datawiz-IN pursues two central priorities: broadening academic and research career opportunities for marginalized students while equipping them with impactful emerging technologies like AI [16]. The program provides:

- Immersive research experiences in biomedical informatics and healthcare AI
- Faculty mentorship tailored to participants’ contexts
- Comprehensive professional development support
- Structured pathways to graduate education and research careers [17]

Building on feedback from our inaugural 2023 cohort, we enhanced the program in 2024 through several key improvements:

- Introduction of biweekly reflection dinners for strengthening mentor–mentee relationships
- Enhanced academic support through GRE preparation workshops and graduate school guidance

- Streamlined administrative processes, including pre-enrollment procedures
- Development of a sophisticated project matching system aligning mentors and mentees
- Expanded professional development opportunities through weekly seminars and research ethics training

This paper evaluates the implementation and outcomes of the Datawiz-IN program across its first two cohorts (2023–2024). We examine:

1. Program structure and implementation strategies, including our approaches to fostering supportive environments and building a sense of belonging through mentorship programs, affinity groups, and mindset interventions
2. Effectiveness of our recruitment and support mechanisms
3. Participant research outcomes in Biomedical AI applications
4. Impact on participants’ technical skills and professional development

Through this analysis, we demonstrate how targeted educational initiatives can successfully promote diversity in AI while advancing healthcare innovations.

## Case study in focus: Datawiz-IN

### Datawiz-IN: NLM R25-funded program

Datawiz-IN represents one component of broader institutional initiatives addressing representation in STEM fields. Through an NIH R25 grant, the program was established to provide research opportunities in biomedical informatics for students from historically underrepresented groups. The program structure combines technical training with mentorship support, offering undergraduate and graduate students an eight-week summer research experience. The curriculum includes both research activities and professional development components, with institutional funding covering participants’ expenses [18, 19].

### Evaluation framework

The program assessment employed a mixed-methods approach examining three primary areas across the 2023 and 2024 cohorts. Demographic data were collected to examine participant representation. Research outputs were analyzed through a systematic review of methodological approaches, data visualization techniques, and potential applications within their respective domains. Additionally, participant experiences were assessed through surveys addressing skill development, mentorship effectiveness, encountered challenges, and overall program perceptions. This evaluation structure was designed to examine the program’s progress toward its stated objectives of expanding participation in biomedical informatics research while supporting participants’ professional development.

## Materials and methods

### Participant recruitment process

Participant recruitment utilized existing institutional networks and partnerships, primarily through established relationships with Minority-Serving Institutions (MSIs). While this approach facilitated outreach to certain underrepresented groups, it may have limited access for potential candidates outside these established networks. The recruitment process would benefit from systematic documentation of outreach methods and response rates to better understand potential selection biases.

The DataWiz-IN pathway program took a holistic approach to recruiting and selecting Scholars. Rather than relying solely on traditional metrics such as college grade point average (GPA) and Graduate Record Examinations (GRE), the program developed strategies to identify broader indicators of potential among students from educationally disadvantaged and underrepresented minority (ED-URM) backgrounds. The recruitment process emphasized identifying research aptitude and interpersonal capabilities that might not have been reflected in conventional academic measures. The program implemented a targeted marketing plan through established community partnerships, building on successful recruitment patterns from IU-MSI SSI, LiFT, and related initiatives. Program faculty activated their professional networks and distributed recruitment materials at biomedical informatics conferences. Additional outreach efforts focused specifically on conferences and venues with higher representation of women and students in technology and biosciences. Candidate evaluation incorporated multiple evidence sources of potential and achievement. Applicants submitted personal statements that addressed their identification with the program’s goals and their potential contributions to broadening participation in biosciences. The selection process considered letters of recommendation from college teachers, counselors, and advisors specifically addressing qualities such as persistence, innovation, collaborative abilities, and problem-solving capabilities in project-based environments. To recognize diverse forms of achievement, the program also accepted recommendations from community leaders documenting candidates’ civic engagement and participation in representation-focused college organizations.

#### Analysis data collection

Initial demographic data collection occurred during the application process, with information stored in the NIH XTrain portal system. While this system enables basic longitudinal tracking of participant metrics, limitations in the standardization of data entry and categorization should be noted. The portal’s predetermined classification systems may not fully capture the complexity of participants’ backgrounds and experiences.

### Project collection and assessment

Project evaluation followed a structured protocol comprising both formative and summative components:

- Regular progress reports submitted to graduate teaching assistants
- Documentation of milestone completion against predetermined metrics
- Assessment of methodological approach and implementation
- Evaluation of deliverables against initial project specifications

Graduate teaching assistants maintained progress documentation, though standardization of monitoring criteria varied across projects. Faculty mentors conducted final evaluations based on project-specific rubrics, with potential variation in assessment criteria across different research domains.

### Survey design and administration

Participant experiences were evaluated through a comprehensive exit survey comprising both structured and open-ended questions. The survey instrument included:

- Eight questions using 4-point Likert scales (Strongly agree, Agree, Disagree, Strongly disagree) assessing:
  - Program learning opportunities
  - Content and structure alignment with career goals
  - Skills confidence
  - Workload satisfaction
  - Performance feedback adequacy
  - Professional competence development
  - Career advancement aspirations
- One mentor evaluation question with a 4-point scale (Exceeded Expectations, Met Expectations, Neutral, Did not meet expectations)
- One overall satisfaction question with a 4-point scale (Extremely satisfied, Somewhat satisfied, Somewhat dissatisfied, Extremely dissatisfied)

- Four open-ended questions capturing:
  - Significant skills and knowledge gained
  - Challenges encountered and potential solutions
  - Plans for implementing gained experience
  - Additional feedback

The survey was administered to all participants (n = 27) at the conclusion of each cohort’s summer experience.

### Data analysis

Program outcomes were analyzed across three key dimensions: representation, innovation, and experience. For demographic analysis, we calculated percentages of participant representation across gender and ethnic categories to demonstrate diversity achievement in both 2023 and 2024 cohorts.

Project outcomes were systematically documented and analyzed through:

- Categorization of technical approaches (e.g., machine learning, genomics, imaging techniques)
- Documentation of healthcare domains addressed (e.g., neuroscience, RNA biology, kidney disease)

- Compilation of institutional representation to demonstrate geographic and institutional representation
- Recording of project deliverables and outcomes

Participant experiences were evaluated through:

- Quantitative analysis of Likert-scale responses (reported as percentages of agreement levels)
- Qualitative analysis of open-ended responses through sentiment analysis using the *syuzhet* R package

The sentiment analysis component generated numerical scores from −1 to + 10 for participant testimonials, acknowledging limitations such as:

- Potential misinterpretation of technical terms in participant feedback
- Limited ability to capture nuanced responses about specific program aspects
- Challenges in accurately scoring complex statements about technical learning experiences

Results are presented primarily through:

- Tabulated project summaries (Tables 1 and 2) showing the range of research approaches and institutional diversity
- Sentiment analysis scores for participant feedback (Table 3)
- Visual representations of participant diversity and selected project outcomes

**Table 1:** 2023 Datawiz-IN Projects

Interns	Project methods and approach	Undergrad Degree	Home Institution
Intern 1	Gene sequence retrieval from NCBI, upstream analysis via UNIPROT, and motif identification using MEME Suite	MS Bioinformatics	IU, Indianapolis, IN
Intern 2	Data integration from CDC and NIH, population data from Census Bureau, with analysis via Python and visualization using histograms, pie charts, and Seaborn heatmaps	BS Biology	North Carolina Agricultural And Technical State University, Kernersville, NC
Intern 3	A pilot study on visual complexity’s effect on cognitive engagement and planned a follow-up for cognitive load in TBI patients	BA Psychology	University of South Florida, St Petersburg, FL
Intern 4	Used machine learning to assess Marion County’s SIDS rates during COVID-19, analyzing social vulnerability, race, and eviction data	BS Biomedical Informatics	IU, Indianapolis, IN
Intern 5	Reviewed 15 RNA modification databases for species representation, biotypes, accessibility, and 2023 updates, including categorization methods	BS Biology	Augustana College, Rock Island, IL
Intern 6	Studied trichostatin-a’s brain impact for AD using gene data from LINCS L1000 and spatial transcriptomics, to pinpoint treatment-affected regions	BS Medical Sciences	University of Cincinnati College of Medicine, Cincinnati, OH
Intern 7	Analyzed CRISPR Cas13 RNA editing in HEK293 cells using RNA-seq, highlighting data quality importance in CRISPR research	BS Biology	Universidad Ana G. Mendez, Carolina, Puerto Rico
Intern 8	Examined delirium biomarkers in ICU patients versus healthy individuals, focusing on muscle and brain indicators, to enhance understanding and treatment approaches	BS Biology	Xavier University Of Louisiana, Thibodaux, LA
Intern 9	Applied machine learning and MRI data analysis to assess BrainAGE as a biomarker for brain aging, linking it to cognitive decline and LMCI risk	BS Chemistry	Purdue University, West Lafayette, IN
Intern 10	Analyzed kidney cellular neighborhoods in CKD and AKI patients using CODEX imaging and Fiji/ImageJ software, to discern structural disease differences	BS Biology	Indiana University, Bloomington, IN
Intern 11	Utilized machine learning and XGBoost for feature selection and impact analysis to identify post-COVID condition patients from EHR data, referencing a rules-based phenotype	BS Electrical Engineering	Texas A and M University, College Station, TX
Intern 12	Investigated DYRK1 A ortholog mbk-1 in C.elegans for Down syndrome research, using motility assays and chemoattraction tests to understand mobility implications	BS Public Health Studies	John Hopkins University, North Plainfield, NJ
Intern 13	Analyzed MMP roles in heart and metabolic diseases using GTeX and AoU data, focusing on tissue associations and demographic impacts, validated by NIH All of Us cohort	MS Bioinformatics	IU, Indianapolis, IN
Intern 14	Reviewed 15 RNA modification databases to analyze and interpret their significance for future molecular biology research	BS Biology	Barry University, Miami Shores, FL

**Table 2:** 2024 Datawiz-IN Projects

Interns	Project methods and approach	Undergrad Degree	Home Institution
Intern 1	Single nucleotide direct RNA sequencing was used to examine m6 A modifications and poly(A) tail lengths in Plasmodium falciparum under heat shock, revealing potential stress response mechanisms	MS Bioinformatics	IU, Indianapolis, IN
Intern 2	MoFNet, a neural network model, was developed to integrate multi-omics data (proteins, genes, and SNPs) to predict Alzheimer’s disease and identify key biomarkers linked to disease progression	BS Computer Science	University of Connecticut, Mansfield, CT
Intern 3	Spatial transcriptomic analysis identified new spatially variable genes (SVGs) in mouse embryos, which were linked to ribosomal functions, cell structure, and potential embryonic lethality	BS Computing and Information	University of Pittsburgh, Pittsburgh, PA
Intern 4	The study analyzed ICU utilization and mortality rates in COVID-19 patients, revealing higher severe outcomes in large metropolitan hospitals compared to non-metropolitan ones	BS Biomedical Informatics	IU, Indianapolis, IN
Intern 5	The study compared diabetes computable phenotypes, revealing social disparities in patient demographics, with DiCAYA having more rural, Hispanic, and unknown race patients than Wells	BS Biomedical Informatics	IU, Indianapolis, IN
Intern 6	The study analyzed structural similarities between 50 RNA modifications and their unmodified bases, uncovering clusters that could enhance RNA biology understanding and aid in targeted therapy development	MS Biomedical Engineering	University of Texas at Dallas, Dallas, TX
Intern 7	The study used accelerometer data to estimate cognitive function in Parkinson’s patients, with Ridge and ElasticNet models performing best	BS Computer Science	Purdue University, West Lafayette, IN
Intern 8	The project identified potential drug candidates targeting RNA modification enzymes, using in silico docking to evaluate binding affinities for FTO, DNMT2, and pseudouridine synthases	MS Bioinformatics	Indiana University, Indianapolis, IN
Intern 9	Mendelian randomization linked elevated MMP11 expression to decreased heart function, highlighting MMP11 as a potential target for cardiovascular therapies	MS Bioinformatics	IU, Indianapolis, IN
Intern 10	he NeoRoo app and NeoWarm device were developed to prevent neonatal hypothermia, demonstrating reduced morbidity and mortality rates in neonates	BS Biology	Albany State University, Albany, GA
Intern 11	The study optimized the HART model for accurate activity recognition, integrating it into a WearOS app to enhance real-time monitoring in healthcare	MS Health Informatics	Indiana University, Indianapolis, IN
Intern 12	This study used single-cell RNA sequencing and R analysis to compare gene expression in Alzheimer’s cells, identifying distinct biomarkers and cell clusters	BS Computer Science	Purdue University, West Lafayette, IN
Intern 13	This study fine-tuned the scGPT model to differentiate Alzheimer’s and healthy brain cells, achieving strong classification results but facing challenges with generalization across diverse datasets	BS Computer Science	IU, Indianapolis, IN

**Table 3:** Sentiment Analysis Results for Program Satisfaction, Skills Confidence, Valuable Learning Opportunities, Content and Structure Alignment, and Professional Competence Development

Category	Min	1 st Qu	Median	Mean	3rd Qu	Max
Overall Program Satisfaction	−0.45	0.075	1.100	2.205	2.200	10.100
Confidence About Skills Gained	0.000	0.800	1.100	0.9909	1.100	2.200
Experienced Valuable Learning Opportunities	0.000	0.800	1.100	1.073	1.100	3.100
Program’s Content Aligned with Career Goals	−0.50	0.250	0.600	0.5545	1.100	1.100
Developed Professional Competence	0.000	0.500	0.500	0.8545	1.100	2.500

## Results

### Outcomes and impact of the program

#### Participant demographics

The Datawiz-IN program’s recruitment efforts focused on increasing participation from historically underrepresented groups in biomedical informatics. Demographic data from the 2023 and 2024 cohorts indicate that women and underrepresented students comprised approximately 60% of participants. While these percentages suggest improved representation compared to typical AI research programs, the limited sample size (*n* = 27 across both cohorts) necessitates caution in drawing broader conclusions about the program’s impact on field-wide representation.

#### Innovations and impacts: spotlight on participant projects

The Datawiz-IN program facilitated impactful AI and data science projects across healthcare domains. Participants leveraged diverse computational approaches, including machine learning, deep learning, and AI-driven analysis techniques, complemented by domain-specific methods such as gene sequencing and spatial transcriptomics (see Table 1 & Table 2). Their work demonstrates how diverse perspectives can enhance AI applications in healthcare, particularly in addressing health disparities and improving patient care for underserved populations.

**Fig. 4:**
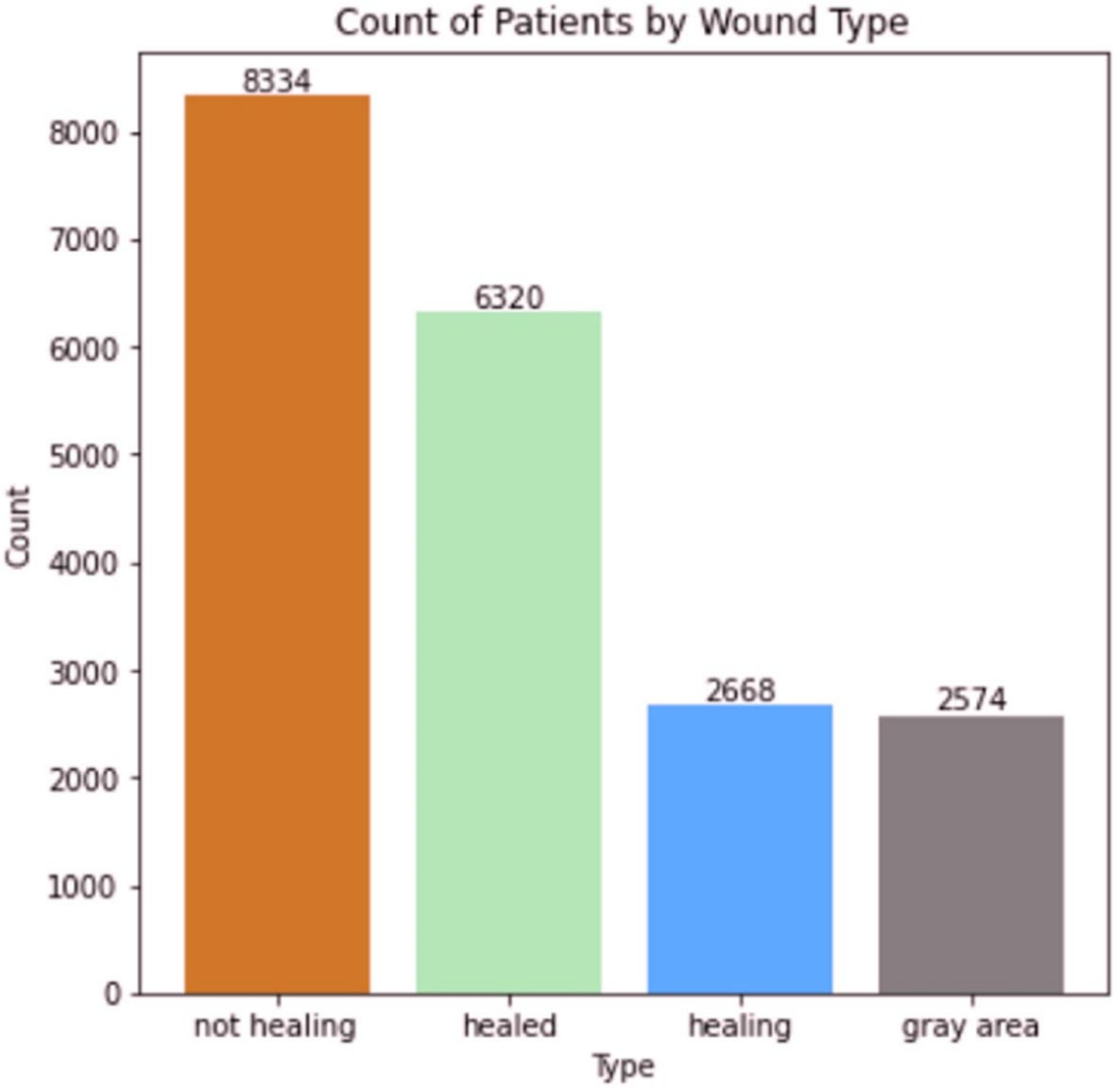
Distribution of Wound Types Across Patients

**Fig. 5:**
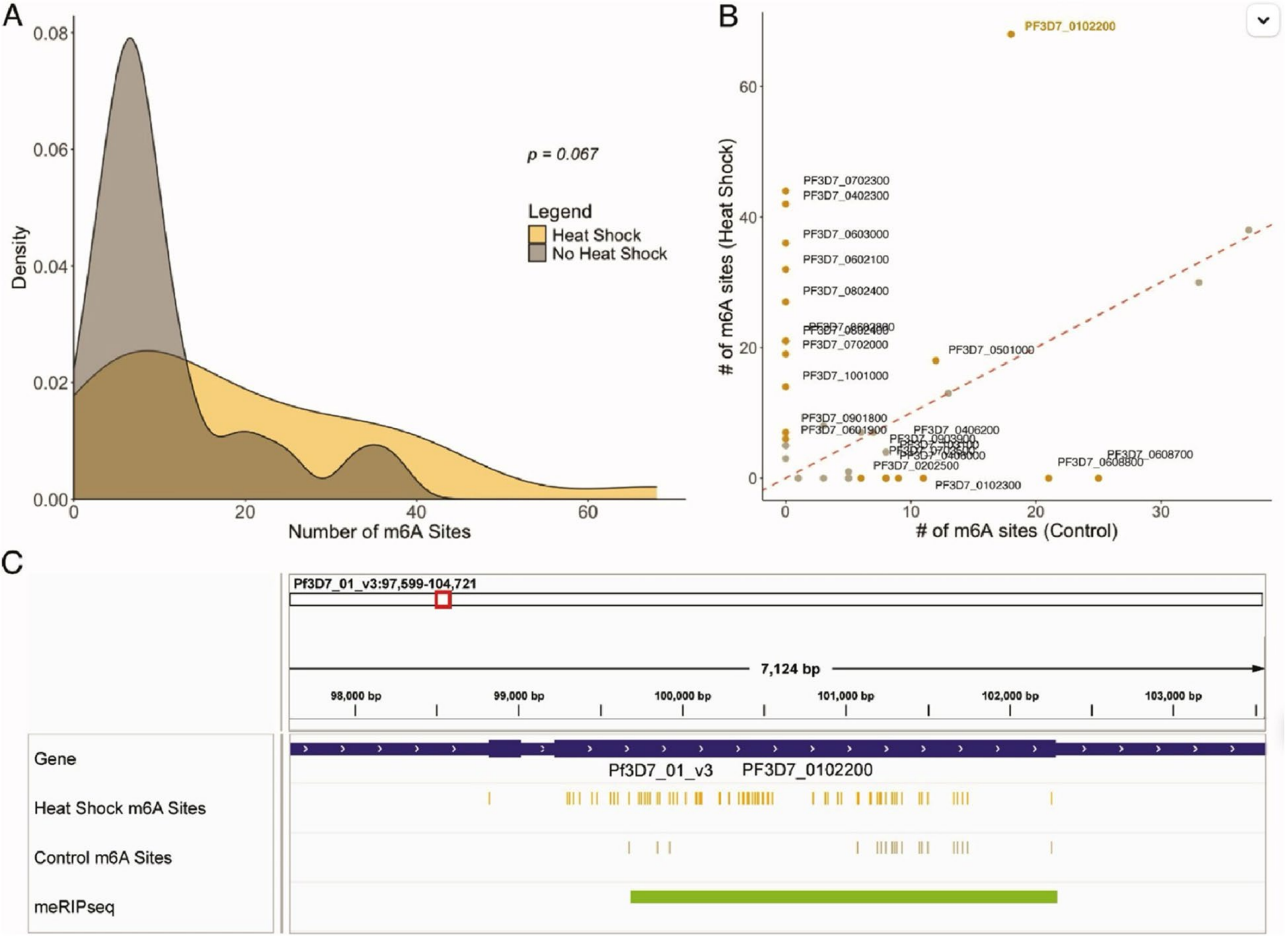
In 2024, a student compared m6 A sites per gene under heat shock and control conditions, finding a higher median in heat shock (16 vs. 8). The scatterplot highlights genes with over 5 additional m6 A sites under heat shock, confirmed by meRIPseq data for PF3D7 0102200 using IGV browser visualization

To illustrate the impact of diverse perspectives in AI research, we highlight two exemplar projects that showcase how participants’ backgrounds influenced their approach to healthcare challenges:

1. AI-Driven Wound Care Classification (Fig. 4): A participant from North Carolina A&T State University developed a machine learning model to address wound care disparities in underserved communities. The project analyzed a dataset of 19,896 patients, creating an automated classification system for wound healing status. The AI model was specifically designed to account for varying wound presentations across different ethnic groups and socioeconomic backgrounds, addressing a known gap in existing wound care algorithms. The visualization shows the distribution of wound types used to train the model, particularly to”gray area” cases (2,574 patients) that often present classification challenges in minority populations.
2. AI-Enhanced Genomic Analysis (Fig. 5): A participant integrated machine learning techniques with genomic analysis to study stress response mechanisms in disease. The project employed deep learning models to analyze m6 A modification patterns, identifying previously unknown stress response genes. The participant’s background in both computer science and biology enabled them to develop an AI approach that could process complex genomic data while maintaining biological interpretability. The visualization demonstrates how AI-driven analysis revealed significant differences in m6 A sites under heat shock conditions (median 16 vs. 8), with the IGV browser validation confirming the AI model’s predictions.

These projects exemplify how Datawiz-IN achieves dual objectives:

- Providing advanced AI and computational training to students from underrepresented backgrounds
- Enabling diverse perspectives to influence the development of AI healthcare solutions

The program’s impact extends beyond technical achievements, as participants bring their lived experiences and cultural understanding to address healthcare challenges through AI applications. This integration of diverse viewpoints with AI expertise has led to more comprehensive and equitable healthcare solutions, as evidenced by the attention to population-specific factors in project designs.

This bar chart illustrates the distribution of wound types in the dataset, including”not healing” (8,334 patients),”healed” (6,320 patients),”healing” (2,668 patients), and”gray area” (2,574 patients).

The dataset highlights the prevalence of non-healing wounds, which are critical for clinical intervention and pose significant challenges in wound care management. These distributions formed the basis for training the machine learning model to classify wound types effectively.

#### Experience evaluation

All 27 participants completed the exit survey, achieving a 100% response rate. The quantitative responses demonstrated strong positive outcomes, with 87% of respondents strongly agreeing that the program provided valuable learning opportunities. The 4-point Likert scale responses (Strongly agree to Strongly disagree) revealed high satisfaction across multiple dimensions, with over 90% of participants reporting significant gains in skills confidence.

Five key themes emerged from the sentiment analysis of open-ended responses, with scores ranging from −1 (most negative) to + 10 (most positive):

- Overall Program Satisfaction (mean score: 2.205)
- Confidence About Skills Gained (mean score: 0.9909)
- Value of Learning Opportunities (mean score: 1.073)
- Career Goals Alignment (mean score: 0.5545)
- Professional Competence Development (mean score: 0.8545)

Qualitative feedback highlighted both program strengths and areas for improvement. One participant emphasized the program’s collaborative environment: Participant 2 quoted’I loved how the fellowship was set up. Meeting so many different people helped boost my confidence’. Technical skill development was frequently mentioned, as illustrated by participant 5:’I gained proficiency in retrieving promoter sequences from UNIPROT…I am confident that these newly acquired skills will greatly contribute to my success and growth’.

Despite the overall positive outcomes, approximately one-third of participants noted challenges related to adapting to new skills, managing time constraints, and balancing personal responsibilities. As participant 11 reflected:’I was new to everything so I had to learn as I went along. Though that was a bit challenging, it paid off’, demonstrating resilience through the learning process.

The sentiment analysis results (Table 3) revealed varying degrees of program effectiveness. While Overall Program Satisfaction showed the highest mean score (2.205) and widest range (−0.45 to 10.100), indicating diverse experiences, consistent positive sentiment was observed in Skills Confidence (mean: 0.9909) and Learning Opportunities (mean: 1.073). The lower mean score for Career Goals Alignment (0.5545) suggests an opportunity for better customization to participant aspirations. Professional Competence Development showed moderate positive sentiment (mean: 0.8545), though with room for enhancement.

## Discussion

This analysis examines the implementation of the Datawiz-IN program in healthcare AI education and research. While the program’s representation of women students and underrepresented participants (approximately 60%) exceeded typical field demographics of 10–15% [6], the small sample size (*n* = 27) limits broader generalizations about the program’s impact on diversity in AI.

The participants’ research projects addressed various healthcare applications, with some projects incorporating demographic considerations:

- The wound care classification project addressed racial and socioeconomic disparities in wound healing assessment
- The diabetes phenotype study revealed important demographic variations in rural and Hispanic populations
- The COVID-19 outcomes analysis identified disparities between metropolitan and non-metropolitan hospitals

Through implementing the Datawiz-IN program, we identified several effective strategies for fostering inclusion in health data science education:

1. Fostering inclusive environments: Creating welcoming spaces for peer interactions and communication has shown positive impact on belonging for marginalized STEM students [20]. Our implementation included inclusive language and active listening practices.
2. Role model mentors: Mentoring connects students to role models with shared identities and experiences, enhancing belonging [21]. Our mentor schemes paired undergraduates with senior STEM students and faculty for both academic and psychosocial support.
3. Affinity groups through cohort-based activities: Cultural sharing activities, including visits to Indianapolis Canal and shared meals, strengthened community bonds.
4. Mindset interventions: Activities addressing belonging uncertainty and stereotype threats improved persistence among women and minorities in STEM [22], helping participants reframe challenges as surmountable.
5. Growth mindset training: Workshops helped students view intellectual abilities as malleable through effort [23], building resilience particularly among negatively stereotyped groups.

Several limitations should be noted. The program’s small scale (14 students in 2023, 13 in 2024) and short duration (8–10 weeks) constrain the generalizability of outcomes. Following approaches used in other NIH-funded programs [24], longitudinal tracking would be necessary to assess career impacts and retention in the field.

Implementation challenges included institutional barriers and resource constraints. While other IU initiatives address similar challenges [25], systematic changes would require broader institutional commitment. The Datawiz-IN program represents one approach to increasing representation in AI research, though its effectiveness compared to alternative interventions remains to be established.

As AI applications in healthcare expand, the need for diverse perspectives in development and implementation grows. While this program suggests potential approaches for supporting underrepresented students in AI research, more extensive studies would be needed to validate these methods. The participants’ projects indicate possibilities for incorporating varied perspectives in healthcare AI development, though their long-term impact on healthcare delivery remains to be determined.

This early-stage initiative provides preliminary insights into supporting diversity in AI education, while acknowledging that sustained efforts across multiple institutional levels would be necessary for systemic change. Future work should focus on rigorous evaluation of intervention effectiveness and the development of scalable, evidence-based support structures.

## Conclusion

This analysis of the Datawiz-IN program offers insights into implementing biomedical data science research experiences for underrepresented students. While our sample size (n = 27 across two cohorts) precludes definitive conclusions, several observations may inform similar initiatives. The program’s approach to recruitment and support structures suggests potential strategies for addressing persistent representation gaps in biomedical data science, though longer-term studies would be needed to validate their effectiveness.

The program’s experience highlights three key considerations for future initiatives: First, recruitment strategies that look beyond traditional academic metrics may help identify promising candidates from diverse backgrounds. However, such approaches require careful validation and refinement to ensure both equity and excellence in selection processes. Second, structured mentorship frameworks combining technical guidance with professional development support appear beneficial, though their optimal implementation may vary by institutional context. Third, integrating discussions of health disparities into technical training may help participants connect their research to broader societal impacts, while maintaining rigorous methodological standards.

## Supplementary Information

Supplementary Material 1.

## Data Availability

Due to the limited sample size, releasing de-identified information raises ethical concerns and risks of reidentification. Therefore, it is not feasible.

## Abbreviations

NLM: National Library of Medicine
NIH: National Insititues of Health
IU-MSI: Indiana University and Minority Serving Institutions
STEM: Science, Technology, Engineering, Mathematics
HBCU: Historically Black college or university
AI: Artificial Intelligence
RNA: Ribonucleic acid
ICU: Intensive Care Unit
IU: Indiana University
IUI: Indiana University Indianapolis
HBCUs: Historically Black Colleges and Universities
UNIPROT: The Universal Protein Resource
MS: Master of Science
BS: Bachelor of Science
