# Supplementary material for: Datawiz-IN: fostering representative innovation in health data science—outcomes from a summer research experience
Source: BMC Med Educ. 2025 May 28;25:793. doi: 10.1186/s12909-025-07298-1 (PMC12121078; doi:10.1186/s12909-025-07298-1)
Supplement: Supplementary file 1 — Supplementary Material 1. [file 12909_2025_7298_MOESM1_ESM.docx]

Supplementary material: Exit survey questionnaire

| **S.No** | **Datawiz End-Of-Program Survey Questions** | **Type** |
| --- | --- | --- |
| 1. | The internship program provided relevant and valuable learning opportunities. | 5-point Likert |
| 2. | The program's content and structure met your expectations and aligned with your career goals. | 5-point Likert |
| 3. | You feel more confident in your skills and abilities after completing the program. | 5-point Likert |
| 4. | Reflecting on your experience, what were the most significant skills and knowledge you gained from the program? (Kindly mention specific details and examples) | Open-ended (avg. 196 words) |
| 5. | How would you evaluate your supervisors'/mentors' availability, capability, and willingness to addressing your questions and/or concerns? | 3-point Likert |
| 6. | The workload over the course of your internship program was satisfactory? | 5-point Likert |
| 7. | Did you receive sufficient feedback on your performance; which in-turn made room for further improvement? | 5-point Likert |
| 8. | The internship program has helped you to develop professional competence | 5-point Likert |
| 9. | You aspire to anticipate career advancements in the same field as your internship program experience. | 5-point Likert |
| 10. | What were some challenges you have encountered during your internship? How could these have been resolved better from your end as well as by the department? | Open-ended (avg. 174 words) |
| 11. | Post your internship program, how do you plan to implement the experience and skills you gained during this program? | Open-ended (avg. 92 words) |
| 12. | Overall, please rate your satisfaction from this internship program | 3-point Likert |
| 13. | Kindly use this text box to provide any further suggestions/feedback/concerns related to your internship program experience. | Open-ended (avg. 33 words) |
